# Clinical parameters in patients with halitosis: a cross-sectional study

**DOI:** 10.3389/fdmed.2024.1427280

**Published:** 2024-08-23

**Authors:** Ahoud Jazzar, Hebah AlDehlawi, Arwa Farag, Sana Alhamed, Sara Akeel, Yasmin Mair, Kenana Flemban, Hidaya Alqassab, Khalid Aljohani

**Affiliations:** ^1^ Department of Oral Diagnostic Sciences, Faculty of Dentistry, King Abdulaziz University, Jeddah, Saudi Arabia; ^2^ Faculty of Dentistry, King AbdulAziz University, Jeddah, Saudi Arabia

**Keywords:** halitosis, oral malodor, stress, Cohen's Perceived Stress Scale, halimeter, salivary cortisol, oral hygiene

## Abstract

**Background:**

Halitosis, a socially and psychologically impactful condition often resulting from oral or systemic issues, is exacerbated by factors like aging, poor oral hygiene, and dietary choices. This study aimed to investigate the association between halitosis and stress by measuring salivary cortisol levels and the Cohen's Perceived Stress Scale (CPSS).

**Methods:**

A cross-sectional study of 40 participants was conducted using questionnaires and clinical measurements to assess halitosis and stress levels. Saliva samples were collected and analyzed for cortisol using ELISA. Participants’ stress was assessed with the Cohen's Perceived Stress Scale Questionnaire (CPSS-10) questionnaire, and halitosis was measured with a Halimeter and self-assessment questionnaire. Clinical measurements included Plaque Index (PI) and the Decayed, Missing, and Filled Teeth (DMFT) score.

**Results:**

Forty subjects were split into a halitosis group (mean age 30.75 ± 10.15) and group with no halitosis (mean age 26 ± 5.3). Objective measures confirmed higher halitosis scores (3.70 ± 0.73) in the halitosis group vs. the second group (2.60 ± 1.67). Compared with the group with no halitosis, the halitosis group exhibited significantly (*p* < 0.05) more plaque (27.92% ± 17.16% vs. 47.50 ± 33.57%; *p* < 0.05) and higher DMFT scores (10.10 ± 2.51 vs. 26 ± 5.30), respectively. Salivary cortisol levels were similar across groups (1.721 ng/ml halitosis, 1.781 ng/ml without halitosis). Correlations showed a moderate positive relationship between DMFT and halimeter scores (*r* = 0.377, *p* = 0.018) and a moderate negative correlation between stress and plaque index (*r* = −0.403, *p* = 0.011), with no correlation between halimeter score and cortisol or CPSS score.

**Conclusions:**

Our findings showed that while halitosis severity correlated with higher DMFT scores and plaque accumulation, there was no significant association with salivary cortisol levels, suggesting that stress, as measured by salivary cortisol, may not be a direct contributor to halitosis. Furthermore, the data suggest that poor oral hygiene is a more significant factor in the development of halitosis than stress levels, as assessed by the CPSS-10.

## 1 Introduction

Halitosis is known as a malodor or foul odor emanating from the oral cavity that is higher than a socially acceptable limit (1). It is ranked as the third most common complaint, after dental caries and periodontal diseases, for which patients seek dental care and advice (2, 3). As stated by the American Dental Association (ADA), around 50% of adults complain of bad breath, of whom 25% are literally suffering from severe chronic halitosis (4). Halitosis not only affects someone's health and well-being but also acts on his social and psychosocial status with a strong impact (3). Despite these major effects that some patients face due to halitosis, they sometimes avoid reporting this problem. This could be due to underestimating the problem or being embarrassed to face it (5).

Halitosis is either genuine, including physiologic like morning breath or pathologic which could be from intraoral or extraoral diseases or pseudo-halitosis, perceived only by the patient and managed with simple hygiene and counseling (3, 6). Volatile sulfur compounds (VSCs) produced by gram-negative bacteria play the most important role in malodor expression (3, 6). It can be detected or measured using either subjective or objective methods. The patient's or other people's own sense of smell is a subjective kind of measuring halitosis. Different clinical methods can be used to objectively measure halitosis; this will include organoleptic measurements, recording of VSC, and microbiological tests, which are the most commonly used methods (4, 7).

In terms of risk factors, old age was found to be significantly associated with increased risk of halitosis. In addition, a significant association was reported between the Decayed, Missing, and Filled Teeth (DMFT) score and halitosis in children. It is typically a result of a combination of factors rather than a single cause. Other factors such as stress can interact and contribute to this condition as well (3, 4). The 10-item Cohen's Perceived Stress Scale (CPSS10) (8) is a useful tool for evaluating psychological stress and has been demonstrated in multiple studies to have sufficient psychometric qualities in terms of validity and reliability across various populations making it a valid and widely acknowledged instrument, which is why it has been translated into numerous languages including Arabic (9). Furthermore, cortisol is a stress hormone detectable in urine, serum, and saliva, considered a potential biomarker for chronic stress (10). Saliva is a site-specific body fluid for halitosis as well as any other oral-related conditions (11). Some studies have shown that anxiety, stress, and depression levels were significantly higher among the self-perceived halitosis group (4). Recent studies indicate a link between stress, depression, and anxiety with levels of cortisol and other biomarkers (12–14). In this study, we aimed to assess the association between halitosis and some of the known risk factors that are linked directly and indirectly; demographics, DMFT, PI and stress to gain a deeper comprehension of the fundamental reasons and constituents of halitosis. Our hypothesis was that stress levels, as measured by salivary cortisol, are associated with halitosis.

## 2 Materials and methods

### 2.1 Study design and settings

The protocol for this study was reviewed and approved by the KAUFD Research Ethics Committee for compliance with the Declaration of Helsinki (15) (ethical approval number: #154-12-22) and conformed to the STROBE protocol (16). The sample size required for this study was calculated to ensure an 80% power to detect a significant association between halitosis and stress, measured by salivary cortisol levels and CPSS. We utilized an expected medium effect size (d = 0.5) based on similar studies examining the relationship between stress and cortisol levels (1). The significance level (alpha) was set at 0.05. The initial calculation indicated that 32 participants per group were needed. To account for potential dropouts and non-compliance, the sample size was increased by approximately 50%, resulting in a final sample size of 46 participants per group.

### 2.2 Inclusion and exclusion criteria

The inclusion criteria were healthy patients who were 18 years old or above. Individuals classified by the American Society of Anesthesiologists (ASA) as ASA 1 with subjective complaints for bad breath who responded to the halitosis questionnaire. We excluded subjects who had recently had antibiotic treatment, smokers, on drugs causing xerostomia, mouth breathers, and those with systemic disease (*n* = 100).

A total of 40 participants (33 females and 7 males) who attended the Comprehensive Care Clinics (CCC) at King Abdulaziz University Dental Hospital (KAUDH) seeking various dental treatments were enrolled over a three-month period. Each participant provided informed consent prior to the study.

### 2.3 Data collection

#### 2.3.1 Halitosis questionnaire

The questionnaire used in this study is validated, and it was adapted from previous studies (17, 18). The original English questionnaire was translated into Arabic using a methodical procedure. Two native Arabic speakers who were also fluent in English were assigned to this process. After each translator completed a written translation, they all convened to review the translation and create a final version. Before the survey was sent, two KAU-FD clinic patients were selected to take part in order to evaluate the questionnaire's face validity. The survey's clarity was also evaluated by the two patients, and their input was taken into account. Two subject-matter experts amended the questionnaire to increase its validity based on the patients feedback. Candidates were approached via a self-assessment halitosis questionnaire using Google Survey Forms®. The 22-item questionnaire included information about demographics, medical history, dietary habits, oral hygiene practice, and dental history.

#### 2.3.2 CPSS-10 questionnaire

CPSS-10 was used to measure the level of stress (19, 20). It consists of two sections: the first includes six negative items measuring the individual perceived stress, while the second consists of four positive items measuring coping (8). Participants were categorized based on their CPSS-10 scores into three stress levels: 0–13 for low stress, 14–26 for moderate stress, and 27–40 for high stress.

### 2.4 Clinical parameters

#### 2.4.1 Intraoral examination and halimeter measurements

The simplified plaque index (PI) was used to evaluate each participant's oral hygiene. The DMFT index was used to evaluate how much caries the subjects had experienced. A Halimeter (Breath Alert, TANITA, Japan) was used to measure the levels of VSC numerically. The values of this device vary from 0 to 5, being: 0 = no odor (normal), 1 = weak odor (normal), 2 = mild odor (normal), 3 = moderate or (perceptible), 4 = strong odor (perceptible), 5 = very strong odor (perceptible). The assessments were done according to the manufacturer's instructions, and the sensor was left at a distance of ∼1 cm from the half-open mouth of the patient. After examining the patients, the air opening was cleaned with a dry cloth, and the Halimeter was gently shaken four to five times in order to remove any moisture and odors that may be left over. The subjects were verbally instructed about the procedure prior to its conduction. Measurements were obtained between 9:00 am and 13:00 pm.

#### 2.4.2 Saliva sample collection

Unstimulated whole-mouth saliva (WMS) samples were collected over a 10-minute period. Samples (5–15 ml) were immediately placed on ice and transferred to the laboratory, where they were processed by centrifugation at 9,500 g for 10 min and stored at −80°C until required. A minimum of 1 h fasting preceded all sample collections, which were undertaken between 09.00 and 13.00 h. Detailed protocols for saliva collection are available (21).

### 2.5 Enzyme-linked immunosorbent assay (ELISA)

Commercially available pre-coated plates (Tecan ELISA kit, Winooski, Vermont, USA) were used. Samples and standards were loaded in duplicate. On a horizontal orbital microplate shaker at room temperature, incubations were performed. All materials were supplied with the kit, and the manufacturer's instructions were followed.

### 2.6 Statistical analysis

All results were exported to Windows® Excel 2019 spreadsheets. The ELISA data were statistically analyzed using IBM SPSS Statistics for Windows, Version 26.0 (IBM Corp., Armonk, NY, USA). The Chi-square test and *t*-test were used to identify the differences between the groups. Spearman's rank correlation coefficient (Spearman's rho) was used to determine the associations between all parameters. Univariate and multivariate logistic regression analyses were conducted to identify predictors of self-reported halitosis, with results reported as odds ratios (OR) and 95% confidence intervals (CI). The significance level was set to *P* < 0.05.

## 3 Results

### 3.1 Demographic characteristics

A total of 40 participants were included. Hundred patients who did not satisfy the criteria were excluded. The mean age was 28.4 ± 8.35 years. Approximately 82.5% of the participants were females with only 17.5% males. Out of the included participants, 50.0% reported oral malodor. There was no significant difference between those with and without oral malodor in terms of the mean age (*p* = 0.327) and sex distribution (*p* = 0.407). Readings are shown in Table 1.

**Table 1:** Demographic characteristics.

Variables		Total	Oral malodor	No oral malodor	*P*-value
Age, years, Mean ± SD		28.4 ± 8.35	30.8 ± 10.2	26.0 ± 5.3	0.327
Sex	Male	7 (17.5%)	5 (25.0%)	2 (10.0%)	0.407
	Female	33 (82.5%)	15 (75.0%)	18 (90.0%)	

### 3.2 Halitosis questionnaire

Among participants with malodor, 57.1% occasionally experienced oral malodor, while only 19.0% frequently suffered from it. Interestingly, 81% indicated they had never been informed of their condition by others, but 19% acknowledged that relatives had noticed it. All 40 participants denied any health issues such as blood, diabetic, cardiac, renal, hepatic, gastrointestinal, sinus, nasal, or throat disorders, and none were on medications, consumed alcohol, or used tobacco products. Nonetheless, 57.1% of participants with malodor reported high sugar consumption, compared to 52.6% in the group without malodor (*p* = 0.775). About 71.5% of the malodor group visit the dental clinic ≤2 times compared to 68.4% of the other group (*p* = 0.801). Moreover, 42.8% of the malodor group brush their teeth more than 2 times per day compared to 47.4% of the other group (*p* = 0.528), as shown in Table 2.

**Table 2:** Results of halitosis questionnaire.

Questions		Malodor	Without malodor	*p*-value
How often do you suffer from halitosis?	Never	1 (4.8%)	19 (100%)	–
	Occasionally	12 (57.1%)	0 (0.0%)	
	Fairly often	4 (19.0%)	0 (0.0%)	
	Very often	4 (19.0%)	0 (0.0%)	
Did someone tell you that you suffer from halitosis?	No	17 (81.0%)	19 (100.0%)	0.045
	Yes	4 (19.0%)	0 (0.0%)	
Do you have blood-related disorders?	No	21 (100%)	19 (100%)	–
	Yes	0 (0.0%)	0 (0.0%)	
Are you diagnosed with diabetes?	No	21 (100%)	19 (100%)	–
	Yes	0 (0.0%)	0 (0.0%)	
Do you take any medication?	No	21 (100%)	19 (100%)	–
	Yes	0 (0.0%)	0 (0.0%)	
Do you complain of any heart-related conditions?	No	21 (100%)	19 (100%)	–
	Yes	0 (0.0%)	0 (0.0%)	
Do you have any gastrointestinal problems?	No	21 (100%)	19 (100%)	–
	Yes	0 (0.0%)	0 (0.0%)	
Do you have any kidney disease?	No	21 (100%)	19 (100%)	–
	Yes	0 (0.0%)	0 (0.0%)	
Do you complain of any liver-related problems?	No	21 (100%)	19 (100%)	–
	Yes	0 (0.0%)	0 (0.0%)	
Do you have any sinuses? nasal or throat problems?	No	21 (100%)	19 (100%)	–
	Yes	0 (0.0%)	0 (0.0%)	
Do you smoke or use tobacco products?	No	21 (100%)	19 (100%)	–
	Yes	0 (0.0%)	0 (0.0%)	
Do you consume alcohol?	No	21 (100%)	19 (100%)	–
	Yes	0 (0.0%)	0 (0.0%)	
Do you consume a high amount of Sugar?	No	12 (57.1%)	10 (52.6%)	0.775
	Yes	9 (42.9%)	9 (47.4%)	
How often do you visit the dentist?	0	1 (4.8%)	2 (10.5%)	0.801
	1	1 (4.8%)	2 (10.5%)	
	2	13 (61.9%)	9 (47.4%)	
	3	3 (14.3%)	4 (21.1%)	
	4	3 (14.3%)	2 (10.5%)	
How often do you brush your teeth?	1	4 (19.0%)	3 (15.8%)	0.528
	2	8 (38.1%)	7 (36.8%)	
	3	7 (33.3%)	4 (21.1%)	
	4	2 (9.5%)	5 (26.3%)	
Do you use other dental products like floss, interdental brush or mouth rinse?	0	7 (33.3%)	3 (15.8%)	0.28
	1	4 (19.0%)	2 (10.5%)	
	2	5 (23.8%)	4 (21.1%)	
	3	3 (14.3%)	3 (15.8%)	
	4	2 (9.5%)	7 (36.8%)	
Do you suffer from dry mouth?	No	21 (100%)	19 (100%)	–
	Yes	0 (0.0%)	0 (0.0%)	
Are you mouth breather?	No	21 (100%)	19 (100%)	–
	Yes	0 (0.0%)	0 (0.0%)	

### 3.3 CPSS-10 questionnaire

Out of the participants, 15% showed low stress, 75.5% had moderate stress, and 12.5% had high stress, with an overall CPSS score of 20.3 ± 6.6. Among those who reported oral malodor, 10% exhibited low stress, 70% moderate, and 20% high, while in those without oral malodor, 20% had low stress, 75% moderate, and 5% high stress, with no statistically significant difference (*p* = 0.286). Similarly, the CPSS score was slightly higher in the oral malodor group compared to those without malodor, with no statistically significant difference (20.6 ± 6.82 vs. 19.9 ± 6.58, *p* = 0.743), respectively. After classifying participants based on their Halimeter score, 23.1% of the participants with ≤2 Halimeter had low stress, 76.9% had moderate stress, and 0.0% had high stress, compared to 11.5% low stress, 69.2% moderate stress, and 19.2% high stress among those with >2 Halimeter, with no statistically significant difference (*p* = 0.265). The CPSS score was slightly higher in the >2 Halimeter group compared to ≤2 Halimeter, with no statistically significant difference (21.3 ± 6.6 vs. 18.5 ± 6.7, *p* = 0.256), respectively. Results are shown in Table 3.

**Table 3:** CPSS scores among the studied participants.

Category	Total	Oral malodor	No malodor absent	*P*-value	Halimeter ≤2	Halimeter >2	*P*-value
Low Stress (%)	6 (15.0%)	2 (10.0%)	4 (20.0%)	0.360	3 (23.1%)	3 (11.5%)	0.265
Moderate Stress (%)	29 (75.5%)	14 (70.0%)	15 (75.0%)		10 (76.9%)	18 (69.2%)	
High Stress (%)	5 (12.5%)	4 (20.0%)	1 (5.0%)		0 (0.0%)	5 (19.2%)	
CPSS Score (Mean ± SD)	20.3 ± 6.6	20.6 ± 6.82	19.9 ± 6.58	0.743	18.5 ± 6.7	21.3 ± 6.6	0.256

CPSS, Cohen's perceived stress scale.

*p* < 0.05.

### 3.4 Clinical parameters and Halimeter measurements

The overall Halimeter score was 3.1 ± 1.4, the DMFT index was 8.3 ± 4.1, and the PI was 34.3 ± 18.2. Among those with oral malodor, the mean Halimeter score was significantly higher than those without oral malodor (3.7 ± 0.7 vs. 2.6 ± 1.7, *p* < 0.001). Similarly, those with oral malodor were associated with significantly higher DMFT index (10.0 ± 2.5 vs. 6.7 ± 4.6, *p* = 0.002) and PI (41.0 ± 17.2 vs. 27.9 ± 17.2, *p* = 0.020), compared with those without oral malodor, respectively as seen in Table 4.

**Table 4:** Halimeter, DMFT index, and PI scores.

Parameters	Total	Oral malodor	No oral malodor	*P*-value
Halimeter Measure	3.1 ± 1.4	3.7 ± 0.7	2.6 ± 1.7	<0.001
DMFT	8.3 ± 4.1	10.0 ± 2.5	6.7 ± 4.6	0.002
PI	34.3 ± 18.2	41.0 ± 17.2	27.9 ± 17.2	0.020

DMFT, decayed, missing, and filled teeth; PI, plaque index.

*p* < 0.001.

### 3.5 Salivary cortisol level (ng/ml)

The mean overall salivary cortisol level was 1.75 ± 1.83 ng/ml. In the comparison between those with and without oral malodor, there was no significant difference (1.72 ± 1.78 vs. 1.78 ± 1.93 ng/ml; *P* = 0.918), respectively. Likewise, the difference between the >2 Halimeter and ≤2 Halimeter groups in terms of salivary cortisol level was not significant (1.84 ± 2.28 vs. 1.77 ± 1.63, *p* = 0.909), respectively.

### 3.6 Study outcomes correlations

The Halimeter measurements showed a weak and non-significant correlation with cortisol levels (*r* = 0.086, *p* = 0.604), CPSS scores (*r* = 0.168, *p* = 0.305), and age (*r* = 0.189, *p* = 0.249). On the other hand, significant weak correlations were observed between Halimeter measurements and DMFT scores (*r* = 0.377, *p* = 0.018) and PI (*r* = 0.350, *p* = 0.029), as shown in Table 5.

**Table 5:** Correlation between study outcomes.

	Cortisol level	CPSS score	Age	DMFT	PI
Halimeter measurement	*r* = 0.086, *p* = 0.604	*r* = 0.168, *p* = 0.305	*r* = 0.189, *p* = 0.249	*r* = 0.377, *p* = 0.018	*r* = 0.350, *p* = 0.029

CPSS, cohen's perceived stress scale; DMFT, decayed, missing, and filled teeth; PI, plaque index.

### 3.7 Predictors of self-reported halitosis

The univariate regression analysis indicated that DMFT was a significant predictor of self-reported halitosis with an OR of 1.26 (95% CI: 1.03–1.54, *P* = 0.023). Similarly, Halimeter measurements were also significant in the univariate analysis, showing an OR of 2.12 (95% CI: 1.03–4.33, *P* = 0.040). In the multivariate regression analysis, DMFT remained a significant predictor with an OR of 1.36 (95% CI: 1.04–1.78, *P* = 0.023), and the PSS (Perceived Stress Scale) score emerged as a significant predictor with an OR of 1.20 (95% CI: 1.01–1.43, *P* = 0.042). The significance of the Halimeter measurements diminished in the multivariate model (OR = 1.37, 95% CI: 0.75–2.47, *P* = 0.300), as shown in Table 6.

**Table 6:** Predictors of self-reported halitosis.

Variables	Univariate regression		Multivariate regression	
	OR	*P*-value	OR	*P*-value
Age	1.07 (0.98–1.17)	0.114	1.10 (0.97–1.25)	0.151
DMFT	**1.26** **(1.03–1.54)**	**0**.**023**	**1.36** (**1.04–1.78)**	**0**.**023**
Halimeter measurement	**2.12** (**1.03–4.33)**	**0**.**040**	1.37 (0.75–2.47)	0.300
PSS score	1.03 (0.94–1.13)	0.539	**1.20** (**1.01–1.43)**	**0**.**042**
Cortisol level	0.95 (0.67–1.34)	0.776	0.92 (0.59–1.43)	0.724
Plaque index	1.04 (0.99–1.07)	0.079	1.00 (0.94–1.07)	0.895

Bold font indicates significant association.

## 4 Discussion

Halitosis, is a condition marked by an unpleasant odor of the mouth. There are a number of potential causes, such as systemic disorders, eating habits, lifestyle variables, and problems with dental health (22). Our objective was to evaluate the relationship between halitosis and several established risk factors, such as demographics, DMFT index, PI, and stress, in a comprehensive approach to understand the multifaceted nature of halitosis. Age can affect halitosis for a number of reasons. Bad breath in the elderly can be caused by a decrease in salivary flow, medications, and chronic illnesses (23). Thus, those factors were considered and excluded. However no difference was detected between those with and without oral malodor in terms of the mean age similar to another study (24). On the other hand, poor dental hygiene or particular food habits could have a greater impact on younger individuals (25). Our results have shown that men had comparable halimeter score compared to women,. This finding contrasts that of a Japanese study where men had higher values of (VSC) compared to women (1). In this study, we evaluated the association between halitosis and stress as one of its indirect contributors.

There aren't many studies in literature that emphasize the connections between halitosis and emotions like anxiety or stress. However, the relationship between anxiety and halitosis has been examined, and clinical observations indicate that anxious circumstances may raise the concentration of VSCs, which in turn causes halitosis (26).

The CPSS-10 questionnaire results suggested no significant differences between groups since the majority of participants in both groups had moderate levels of stress, which have the range of 14–26 points in total on the CPSS-10 scoring system. After classifying patients according to halimeter score, the CPSS score was slightly higher in the >2 Halimeter group compared to ≤2 Halimeter, with no statistically significant difference. Additionally, there were no significant differences among genders in the responses to the CPSS-10 questionnaire. Kato and colleagues reported that individuals who worry about halitosis have shown a higher level of psychological stress compared to those who do not, regardless of the presence of genuine halitosis. Suggesting that these individuals will require psychological treatment besides dental treatment (27). In agreement with another study that suggested that patients who were affected by halitosis can have a higher rate of developing clinical depression and, thus, higher levels of stress and stress hormones (28).

Regarding salivary cortisol levels, this study's results showed no significant difference between patients who complain of halitosis and patients with no complaint of oral malodor. The majority of participants had normal levels of salivary cortisol when compared to the normal range of salivary cortisol at the collection time of each participant's sample individually. Similarly, a Japanese study by kato et al. that measured chromogranin A and cortisol levels in the saliva of patients complaining of oral malodor also showed no significant differences in the cortisol levels of both groups (27). On the other hand, another study used the Cornell Medical Index (CMI) Health Questionnaire to assess the tendency toward neurosis. They correlated the levels of salivary stress markers and the categories of the CMI questionnaire, revealing that the cortisol levels were higher in the group with a tendency towards neurosis compared to the normal group (1).

The results of our study also revealed higher scores of DMFT and plaque indices in patients complaining of oral. A high DMFT score is indicative of poor oral health, which can be a major cause of halitosis. Dental cavities and decaying teeth can host bacteria that can cause an increase in VSCs inside the oral cavity of the patient. In a similar vein, gaps left by missing or poorly filled teeth can harbor bacteria and food particles, which can cause bad breath (26). A significant positive correlation was found between the halimeter measurement and the DMFT index. This finding aligns with that of Anbari et al., who reported a statistically significant association between DMFT and halitosis (29). On the other hand, Evirgen et al. showed that the DMFT score was comparable in those with or without halitosis (30). The differing results may be attributed to their study population consisting of dental students who likely have better oral hygiene practices and dental health awareness. Moreover, the observed negative correlation between perceived stress, as measured by the CPSS scores, and indicators of oral health such as DMFT and plaque index presents an intriguing aspect of our findings that invites a multifaceted interpretation.One explanation for this counterintuitive association is that stressed individuals may practice better oral hygiene as a coping mechanism or due to increased health awareness. Alternatively, stress might change saliva composition, enhancing oral defenses. This suggests that stress can have varied effects on dental health beyond the usual negative impacts. Further research is warranted to elucidate the underlying mechanisms and to determine whether these patterns hold across broader populations.

The regression analysis indicates that DMFT and perceived stress (measured by the PSS score) are associated with self-reported halitosis. The OR for DMFT suggests that individuals with higher dental caries experience are more likely to report halitosis. However, the significance of Halimeter measurements diminishes when considering other factors in the multivariate model. These findings align with previous research that highlights the multifactorial nature of halitosis, involving both oral and psychological factors (4). Dental professionals should consider these associations when assessing and managing halitosis in clinical practice.

The limitations of this paper include the need for longitudinal studies in order to assess the correlation of halitosis and salivary stress biomarkers, taking into account the fluctuations of salivary cortisol levels, salivary flow rate should have been taken into account that might affect the measured cortisol levels, although investigators were aware of the normal flow rates that were perceived but variations should have been investigated in addition to a larger population for the sampling to be conducted in a manner that can reveal a significant correlation between the two if present. A definitive conclusion would also be more challenging to reach due to the unequal sample sizes between the male and female participants.

## 5 Conclusion

Our study found a significant correlation between halitosis severity and higher DMFT scores, as well as increased plaque accumulation, indicating that poor oral hygiene is a major contributor to halitosis. There was no significant association between salivary cortisol levels and halitosis, suggesting that stress, as measured by salivary cortisol, may not be a direct contributor to halitosis. However, the PSS score emerged as a significant predictor in the multivariate analysis, indicating that perceived stress levels might indirectly influence oral hygiene behaviors and consequently halitosis. These results underscore the importance of maintaining good oral hygiene in managing halitosis and suggest that psychological factors like stress might influence oral health indirectly by affecting hygiene practices.

## Data Availability

The original contributions presented in the study are included in the article/Supplementary Material, further inquiries can be directed to the corresponding author.
