# Supplementary material for: Clinical parameters in patients with halitosis: a cross-sectional study
Source: Front Dent Med. 2024 Aug 23;5:1427280. doi: 10.3389/fdmed.2024.1427280 (PMC11797787; doi:10.3389/fdmed.2024.1427280)
Supplement: Supplementary file 1 [file Datasheet1.pdf]

Halitosis questionnaire

البيانات الشخصيةDemographic Data

العمر Age:

الجنس Gender:

الوظيفة Occupation:

**Halitosis Specific Questions**<br>**أسئلة متعلقة برائحة الفم الكريهة**		
1.Do you suffer from halitosis?<br>هل تعاني من رائحة فهم كريهة؟	**Yes**<br>**نعم**	**No**<br>**لا**

2.How often do you suffer from halitosis?<br>كم مرة تعاني من رائحة فم كريهة؟	**0**	**1**	**2**	**3**	**4**

3.Did someone tell you that you suffer from halitosis?<br>هل أخبرك أحدهم أنك تعاني من رائحة فم كريهة؟	**Yes**<br>**نعم**	**No**<br>**لا**

**Medical History Questions**<br>**أسئلة متعلقة بالتاريخ المرضي**	**Yes**<br>**نعم**	**No**<br>**لا**
1. Do you have blood related disorders? (if yes, specify) ……………………..<br>هل تعاني من أمراض متعلقة بالدم؟ (وضح ما هي في حال الإجابة بنعم)		
2. Are you diagnosed with diabetes? (if yes, specify) ……………………..<br>هل تم تشخيصك بمرض السكري؟(وضح النوع في حال الإجابة بنعم)		
3. Do you take any medication? (if yes, specify) ……………………..<br>هل تستخدم أي أدوية؟ (اذكر اسم الدواء في حال الإجابة بنعم)		
4. Do you complain of any heart related condition?<br>هل تشتكي من أي مشاكل لها علاقة بالقلب؟		
5. Do you have any gastrointestinal problem?<br>هل لديك مشاكل في الجهاز الهضمي؟		
6. Do you have any kidney disease?<br>هل لديك أي أمراض في الكلى؟		
7. Do you complain of any liver related problems?<br>هل تشتكي من أي مشاكل في الكبد؟		
8. Do you have any sinus, nasal or throat problems?<br>هل لديك أي مشاكل في الأنف، الجيوب الأنفية أوالحلق؟		
**Diet and Habits**<br>**العادات والتغذية**	**Yes**<br>**نعم**	**No**<br>**لا**
1. Do you smoke? (if yes, how often?) ……………………..<br>هل أنت مدخن؟ (كم عادة ؟إذا كان الجواب نعم)		
2.Do you consume alcohol? (if yes, how often?) ……………………..<br>هل تتناول الكحول؟ (كم عادة؟ إذا كان الجواب نعم)		

3.Do you consume high amount of sugar?<br>هل تتناول كميات كبيرة من السكريات؟		

**Dental and Oral Hygiene Questions**<br>**العناية بالفم والأسنان**					
1.How often do you visit the dentist?<br>كم عادة تزور طبيب الأسنان؟	**0**	**1**	**2**	**3**	**4**
2.How often do you brush your teeth?<br>كم عادة تفرش أسنانك؟	**0**	**1**	**2**	**3**	**4**

	**Yes**<br>**نعم**	**No**<br>**لا**
3. Do you use other dental products like floss, interdental brush, or mouth rinse?<br>هل تستخدم أدوات تنظيف أسنان أخرى؟ (خيط الأسنان، فرشاة ما بين الأسنان، غسول للفم)		
4.Do you suffer from dry mouth<br>هل تعاني من جفاف في الفم؟		
5.Are you mouth breather?<br>هل تتنفس عن طريق الفم؟		

Perceived Stress Scale (PSS)

مقياس الشعور بالإرهاق

The questions in this scale ask you about your feelings and thoughts during THE LAST MONTH. In each case, you will be asked to indicate your response by placing an “X” over the circle representing HOW OFTEN you felt or thought a certain way. Although some of the questions are similar, there are differences between them and you should treat each one as a separate question. The best approach is to answer fairly quickly. That is, don’t try to count up the number of times you felt a particular way, but rather indicate the alternative that seems like a reasonable estimate.

0=Never 1=Almost Never 2= Sometimes 3=Fairly Often 4=Very Often

الأسئلة في هذا الاستبيان تتعلق بأحاسيسك وأفكارك خلال الشهر الماضي. ويطلب منك في كل سؤال أن تبين كم مرة أحسست أو فكرت بطريقة معينة. وإن كانت بعض الأسئلة متشابهة، غير أن هناك اختلافات بينها، لذلك المرجو منك أن تتعامل مع كل سؤال على أساس أنه سؤال مستقل. والطريقة المثلى هي أن تجيب على كل سؤال بسرعة، أي ألا تحاول أن تحسب بالضبط عدد المرات التي أحسست بشيء معين، بل أن تجيب على السؤال بتقدير معقول. للإجابة على كل سؤال من الأسئلة التالية، اختر اجابة واحدة.

0=لم يحدث أبداً 1=تقريباً لم يحدث أبداً 2=أحياناً 3 =في كثير من الأحيان 4=كثيراً جداً

Cohen, S., Kamarck, T., & Mermelstein, R. (1983). A global measure of perceived stress. Journal of Health and Social Behaviour, 24, 385-396. Reprinted with permission of The American Sociological Association

**Perceived Stress Scale (PSS)**<br>**مقياس الشعور بالإرهاق**	**0**	**1**	**2**	**3**	**4**
1.In the last month, how often have you been upset because of something that happened unexpectedly?<br>خلال الشهر الماضي كم مرة غضبت بسبب حدث غير متوقع؟					
2.In the last month, how often have you felt that you were unable to control the important things in your life?<br>خلال الشهر الماضي كم مرة شعرت بأنك لا تستطيع السيطرة على الأمور المهمة في حياتك؟					
3. In the last month, how often have you felt nervous and stressed?<br>خلال الشهر الماضي كم مرة شعرت بأنك متوتر أو مضغوط؟					
4.In the last month, how often have you felt confident about your ability to handle your personal problems?<br>خلال الشهر الماضي كم مرة شعرت بالثقة في قدرتك على تخطي مشاكلك الشخصية؟					
5. In the last month, how often have you felt that things were going your way?<br>خلال الشهر الماضي كم مرة شعرت أن الأمور تسير في حياتك كما تريد؟					
6. In the last month, how often have you found that you could not cope with all the things that you had to do?<br>خلال الشهر الماضي كم مرة وجدت أنك غير قادر على التعامل مع كل ما يجب عليك القيام به؟					
7. In the last month, how often have you been able to control irritations in your life?<br>خلال الشهر الماضي كم مرة استطعت السيطرة على المشوشات في حياتك؟					
8. In the last month, how often have you felt that you were on top of things?<br>خلال الشهر الماضي كم مرة شعرت أنك مسيطر على حياتك؟					
9. In the last month, how often have you been angered because of things that were outside your control?<br>خلال الشهر الماضي كم مرة شعرت بالغضب بسبب أمور لم تكن تحت سيطرتك؟					
10. In the last month, how often have you felt difficulties were piling up so high that you could not overcome them?<br>خلال الشهر الماضي كم مرة شعرت بتراكم الصعوبات لدرجة لم تمكنك من تخطيها؟					
